# Infrared Thermography of Teat in French Dairy Alpine Goats: A Promising Tool to Study Animal–Machine Interaction during Milking but Not to Detect Mastitis

**DOI:** 10.3390/ani14060882

**Published:** 2024-03-13

**Authors:** Pierre-Guy Marnet, Alejandro B. Velasquez, Alen Dzidic

**Affiliations:** 1 Department of Animal and Food Sciences, Institut Agro Rennes-Angers, F-35042 Rennes, France; 2 UMR SELMET, CIRAD, INRAe, Institut Agro, F-34398 Montpellier, France; 3 Department of Agricultural Sciences and Aquaculture, Faculty of Natural Resources, Universidad Católica de Temuco, Temuco 4780000, Chile; avelasquez@uct.cl; 4 University of Zagreb Faculty of Agriculture, 10000 Zagreb, Croatia; adzidic@agr.hr

**Keywords:** goat, IRT, thermography, SCC, mastitis, machine milking, teat shape, udder balance

## Abstract

**Simple Summary:**

Infrared thermography (IRT) is a non-invasive technology that is of interest both for diagnosing mastitis and describing possible interactions between milking machine liners and teat tissue in cows. Very little was known about these applications in dairy goats, although this species is increasingly affected by mastitis, which is not only of infectious origin. This study aims to fill this gap by investigating the thermal responses of teats to the milking machine in goats with different levels of udder inflammation. IRT fails to detect mastitis early in goats and cannot be used for prophylactic purposes in goats. IRT measurements were influenced by milking, and the results differed between unbalanced glands and different teat shapes, indicating differences in the interaction of the machine with the teat tissue. The IRT, therefore, appears to be a good instrument for measuring the effects of the milking machine. In the future, it could help to better adapt the machine equipment and settings to the animals and improve the efficiency and well-being of the animals.

**Abstract:**

There is a need to develop tools for mastitis management in goats and to measure the effects of milking machines on teats. Infrared thermography (IRT), as shown in cows, was a good candidate for early mastitis detection and focusing on milking equipment and settings implicated in potential problems. The aim of this study was to test IRT to detect udder inflammation and the effects of mechanical milking on teats in relation to inflammation status, udder balance, and teat shape in Alpine goats. IRT spectra were compared before and after milking in 551 goats from three commercial herds compared to their individual SCC (somatic cell count). We found no regression or trend between logSCC and IRT measurement or response to milking, even in highly inflamed goat udders. The effect of milking was significant (*p* < 0.05) with global temperature reduction after milking, but differences were seen between teat parts and unbalanced half udders. The highest reduction in skin temperature was observed at the teat orifice (−1.06 ± 0.05) and the lowest at the teat barrel (−0.37 ± 0.05). The teats with long barrels showed more IRT reactions, which clearly indicates poor adaptation to the liners used. In conclusion, the IRT was not able to detect mastitis, but it is a good tool to diagnose the effects of the milking machine in order to adapt milking equipment and settings to the goats and improve their welfare.

## 1. Introduction

Infrared thermography (IRT) is a well-known technology. Used for the first time in human medicine on a living organism, it enables the detection of tissue infrared emission, which is largely dependent on the tissue’s underlying blood flow and metabolic rate [1,2].

Its use in mastitis detection is well documented, as local hyperemia occurs at the udder level during inflammation [3]. The ability of IRT to detect artificially induced mastitis or inflammation following toxic challenges or bacterial artificial infection with E-coli has been confirmed [4,5,6]. Other work on cows [7,8,9], on crossbred Karan Fries (*bos Taurus* × *bos indicus*) [10], and on camels [11] suggested a possible use of IRT for early diagnosis of natural subclinical mastitis, as strong to moderately significant (*p* < 0.05) positive regression between udder surface temperature and SCC (somatic cell count). Nevertheless, it is sometimes reported that clinical signs were present before the thermal reaction of the udder [12]. Sometimes, the IRT did not show better sensitivity and precision than the CMT (California Mastitis Test) [13], and some authors find it difficult to differentiate between healthy and inflamed quarters with the IRT [5], suggesting that it is difficult to use the IRT for the detection of early signs of mastitis. In dairy sheep, the conclusions for IRT diagnosis of mastitis are the same. While the triggering of acute mastitis by bacteria or toxin infusion was easily confirmed by IRT, the detection of natural and chronic mastitis was more difficult, even when clinical signs were recognizable [14]. This could be due to endogenous reaction cycles to the infection and local edema, which could reduce blood flow locally and lead to a biased diagnosis [3]. Previous studies on IRT of udders and teats [15] do not always distinguish between healthy and infected glands, depending on the point (or area) at which temperature was measured on teats and cisterns. This point is important because when IRT was applied to the teat surface rather than the cow’s udder, the authors [16] found a better mastitis prognosis and higher correlations between IRT values at the teat end and SCC and CMT scores. In addition, a significant (*p* < 0.05) difference in udder shape (more or less pendulous), udder skin wall thickness, and teat thickness (higher in cows than in goats) could explain these difficulties and possible interactions of environmental conditions and skin temperature in small ruminants. The higher SCC found in goats varies within a day [17] and may not always be related to intramammary bacterial infections. In addition, high SCC levels in goats could be due, at least to some extent, to co-infection with lentivirus and non-infectious inflammatory factors such as parity, stage of lactation, season, oestrus, milking rhythm, and response to machine milking [18,19,20,21,22,23,24,25]. It is assumed that these factors mask the connection between SCC and bacterial infection of the udder. It is assumed that the distribution of SCC overlaps considerably between infected and uninfected animals [26] and consequently could also distort the correlations between SCC and IRT. 

Therefore, the IRT could be used for a more efficient study of the direct effects of the milking machine at the teat level. The changes in teat fluid circulation caused by machine milking in cattle increase teat skin temperature between 0.8 and 2.1 °C [27,28,29,30]. This increase may be due to circulatory changes in the teat wall caused by the mechanical friction of the teat in the liners [27], by hot milk flowing through the colder teat cistern, by a reduced ability of the teat to dissipate surface heat when they are inside the liners, and finally by a reaction of the cutaneous vascular plexus and an increase in blood flow (active hyperemia) [28]. Kunc et al. [31] were the first to study the effects of milking equipment on cows. They showed a different effect of tubular or triangular liners and decreased teat temperature after milking with increasing vacuum. A significant (*p* < 0.05) drop in temperature after cleaning the cow’s teats and an increase during milking, particularly in the middle part of the teat, were strongly influenced by the liner pressure during the massage phase, especially during over-milking [29]. These authors showed that the buckling pressure of the liner has a considerable influence on the IRT of the teat skin by changing the pressure exerted on the teat (extended liners increase the teat temperature more than soft liners). In dairy sheep, IRT was also used to evaluate the effects of different vacuum levels on the teats. Immediately after milking, the results showed a general decrease in teat temperatures, particularly at higher vacuum levels, with differences between the teat end and base [32]. Different effects of milking have been reported in goats at different positions on the teat, considering that there is a global increase in teat skin temperature after milking [15]. The same authors found no interaction with the health status of the udder in the middle of the teat and a negative effect of teat wall thickness on temperature. On the contrary, our own preliminary trial in goats showed a reduction in teat skin temperature after machine milking [33].

Moreover, most of these studies were conducted on a small number of animals and in experimental facilities with well-controlled working conditions. In this context, the aim of this study was, firstly, to evaluate the influence of the milking machine on teat temperature using IRT in French Alpine goats from whole herds on different commercial farms with different milking equipment and management; secondly, to analyze the influence of udder imbalance and teat shape using IRT and, thirdly, to verify whether IRT in dairy goats is suitable for evaluating the degree of inflammation as part of better mastitis management.

## 2. Materials and Methods

### 2.1. Farms and Animals

This study was carried out on three different farms in the Brittany region of France, all keeping an Alpine breed of goat. One farm has ecological (organic) management with grazing and low supplementary feeding (101 dairy goats), which supplies the dairy plant. There are two other farms on which the study was carried out with goats in the barn in classical housing (220 and 234 dairy goats) with a ration of hay, production concentrates, vitamins, and minerals). Moreover, milk was delivered to the milking plant. All have side-by-side milking parlors with a low milk line, but equipment of different brands (1 Delaval of 2 × 12, 1 Boumatic/Gascogne Melotte of 2 × 30, 1 Fullwood-Packo of 2 × 30). Apart from the Delaval parlor, the other two have had the same cluster (liners, cups, tubes, and fullwood-type claw). All had shut-off valves at the base of the teat cup (Delaval) or on each short milk tube (Fullwood), which open automatically when the liner is connected to the teat and close when they are removed or if air suddenly enters the liner. The Delaval milking parlor and the Boumatic/GM milking parlor were equipped with a vacuum shut-off system without the cluster remover (farmers did not know the milk flow thresholds-around 150–200 g/min as originally recommended by manufacturers or local distributors), and the remaining milking parlor had neither vacuum shut-off system nor the cluster remover. The milking systems on all farms were inspected annually by an independent organization. The machine settings were similar on all farms, with 37 or 38 KPa milking vacuums at 80 p/min and a 60/40 ratio.

### 2.2. Animal Recording

#### 2.2.1. Milk Quality Measurements

We collected the individual milk quality control results for the 551 goats (milk production and SCC) on the closest day after the day of our own measurement on the three farms (7, 8, and 10 days after the IRT measurements).

#### 2.2.2. Udder and Teat Shapes Scoring

**Figure 1:**
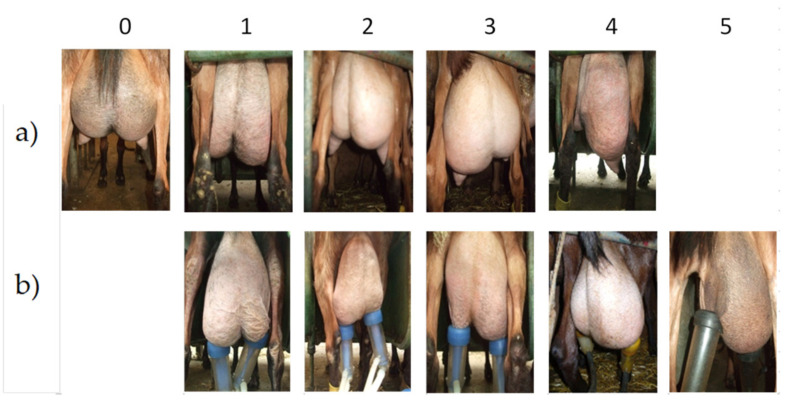
Evaluation of the degree of morphological imbalance of the udder (**a**), i.e., before milking, and functional imbalance (**b**), i.e., during milking, between the two half-mammary glands. The degree of imbalance can vary from 0 (balanced udder) to 1 to 5, depending on how severe the imbalance is. During milking the udder is never balanced, and before milking the degree of imbalance is never 5.

**Figure 2:**
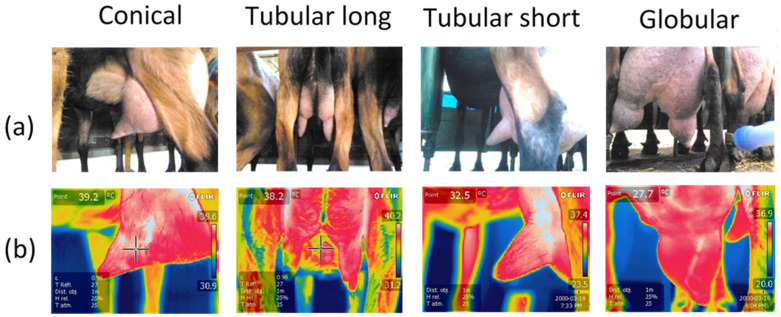
Classification of teat shapes on classic digital photos (**a**) and IRT images (**b**) into four classes (conical, tubular long, tubular short, and globular).

Using digital photos of their udders (Sony Cybershot- DSC-HX50V, Sony, Chomburi, Thailand), the 551 goats were classified according to their degree of unbalance before milking (morphological unbalance) on a 6-point scale (0 balanced and 1 to 5 for increasing unbalance). The imbalances observed during milking called functional imbalance (one half of the udder empties faster than the other) were also graded on a similar scale from 0 to 6 (Figure 1). Photographs of the teats were taken, and the teat shapes were divided into four classes (conical, tubular long, tubular short, and globular) (Figure 2).

#### 2.2.3. IRT Recording 

IRT of the two teats per goat was performed shortly before milking and shortly after milking and cluster removal using a Flir E-60 camera (320 × 240 pixels, automatic calibration, and temperature correction, <0.05 °C thermal sensitivity, −20 °C to 120 °C temperature range, 60 Hz frame rate, accuracy +/−2% within the ambient temperature range of +10 °C to 35 °C). Thermal images (n = 1300) were analyzed using the ThermaCam Researcher Pro 2.10 software (FLIR Systems Inc., Wil-sonville, OR, USA).

**Figure 3:**
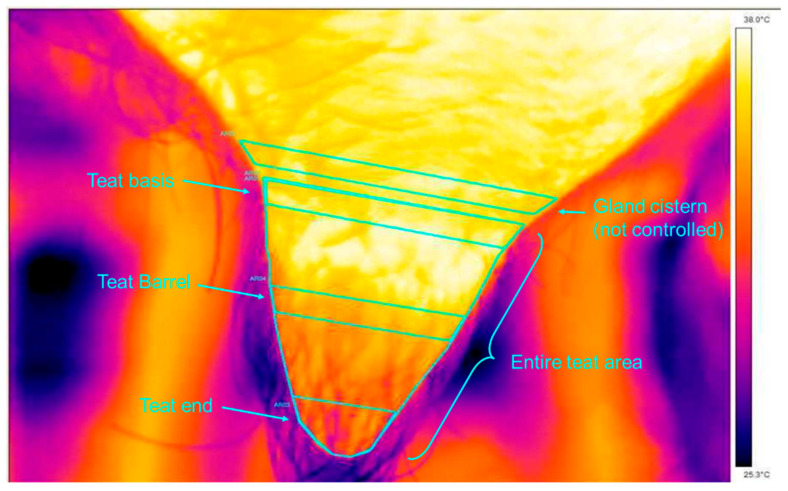
Different teat areas assessed with the IRT.

The methodology followed what was described by Alejandro et al. [15] and four areas per teat were defined: the mean temperature on a strip of 1 cm drawn exactly at the base of the teat (a numerical image associated with the thermal image helped us to determine the connection between the teat and the cistern of the gland), as well as a zone of interaction between the teat and the mouthpiece lip of the teat liner (teat base). The mean temperature was also measured in a similar strip of 1 cm at the far distal part of the teat (teat end), as this area of the teat sphincter is potentially more affected by the milking vacuum. The mean temperature was also measured on a last strip of 1 cm in the middle of the teat (teat barrel), as this zone is better massaged during liner buckling. A final measurement was carried out on the entire teat surface (total teat area) (Figure 3). All these measurements were taken before and after milking for each teat during morning and evening milking.

### 2.3. Statistical Analysis

A total of 4408 complete data sets (551 goats × 2 halves udder × 2 milking per day × 2 (before and after milking)) were obtained, but only 4406 usable IRT images were obtained due to blurred or poorly referenced images. 

The MIXED procedure of SAS 9.4 for Windows (SAS Institute Inc., Cary, NC, USA) with a mixed model was used for the statistical analysis. The mixed model for the dependent variable temperature difference (temperature after−temperature before) included the fixed effects of teat shape (levels: conical, globular, tubular short, and tubular long), the effect of teat area (levels: teat base, teat barrel, teat end, and total teat area), udder balance (levels: unbalanced, balanced morphologically, and functionally), parity (levels: first, second, third, or more) and their double interactions. The random effect was goats within the farm. The influence of farm and milking timing was originally included in the model, but without significant effect and improvement of our model, they were not included in the final model. Milk production was added as a covariate, the Kenward–Roger covariance matrix was adjusted, and the goat within the farm was considered a subject for repeated measurement. The Tukey–Kramer test was used to analyze pairwise differences in least-square means (LS Means). Effects were defined as significant if *p* < 0.05. The model for the dependent variable logSCC contained fixed effects of teat shape (level: conical, globular, tubular short, and tubular long), parity (levels: first, second, third, or more), and farms (levels: 1, 2, and 3). The effect of udder balance was originally included in the model, but without significant impact and improvement to our model, it was not included in the final model. The linear regression with dependent variables (temperature difference and temperature before) and independent variable (logSCC) was used for the statistical analysis in the REG procedure of SAS 9.4 for Windows (SAS Institute Inc., Cary, NC, USA). 

All procedures performed on animals were authorized in accordance with French regulations (decree no. 2001-464; 29 May 2001; https://www.legifrance.gouv.fr/eli/decret/2001/5/29/AGRG0001697D/jo/texte (accessed on 6 May 2023)).

## 3. Results

### 3.1. Effect of Milking on IRT

The three farms did not differ significantly (*p* > 0.05) in mean teat temperature before and after milking nor in temperature differences in response to machine milking. Milk yield significantly influenced (*p* < 0.05) the milking-induced change in teat temperature.

The time of milking did not affect significantly (*p* > 0.05) the teat temperature difference, with a higher value in the afternoon than in the morning (−0.69 ± 0.11 °C and −0.66 ± 0.06 °C, respectively).

The frequency of the different teat shapes was similar between farms: 30.29 % were conical, 28.26 % cylindrical with a short barrel (tubular short), 10.91 % cylindrical with a long barrel (tubular long), and 30.54 % globular. 

While teat shape had no effect on this overall response (*p* = 0.73), the effects of teat area (Table 1) and the interaction between teat shape and teat area were significantly different (*p* < 0.05) (Table 2). 

The influence of parity and interactions between parity and teat areas on the effects of milking on teat IRT was highly significant (*p* < 0.05). In the older goats, we recorded a smaller drop in temperature after milking (Table 3). This effect varied depending on the teat area, with teat skin temperature after milking at teat barrel level being higher on average in older goats compared to younger goats (Table 3).

The incidence of unbalanced udders was similar between farms and did not differ between parities of goats. Of the 1102 udders halves and teats examined, 62.65% were correctly balanced and 37.35% functionally unbalanced with an average score of 2.5 (of which 55% were morphologically unbalanced before milking with an average score of 1.33). Imbalance scoring had no effect on the thermal response of the teats, and it must be emphasized that the number of goats per scoring class for imbalanced glands varied widely (from 1.6% to 11.8% of the total number of glands scored for imbalanced classes).

Nevertheless, the average teat temperature difference in response to milking did not differ between goats with unbalanced and well-balanced udders (−0.65 ± 0.07 °C and −0.68 ± 0.06 °C, respectively, *p* = 0.68), but the effects of milking appeared to differ depending on the teat area studied (Table 4).

### 3.2. Udder Inflammation and Regression between IRT and Mean LogSCC

Farm, teat shapes, and parity influenced the average individual logSCC (*p* < 0.05) (Table 5). 

The globular teats had a higher mean log SCC, while the tubular long teats had the lowest log SCC. Parity effects showed a significantly (*p* < 0.05) lower SCC in the first lactation, then in the second or third lactation, and in the more important parities.

**Figure 4:**
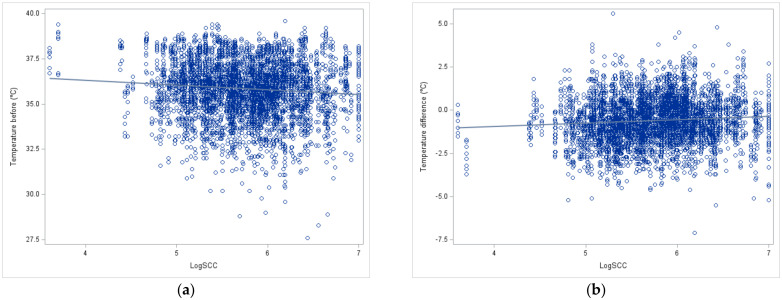
Linear regression between log SCC and IRT measurement before milking (**a**) and difference of IRT measurement (after-before) milking in goats (**b**).

The regression between teat surface temperature before milking and temperature difference (after minus before milking) with udder inflammation (log SCC) was significant (*p* < 0.05) but very low (b1 = −0.28 and +0.19, respectively) (Figure 4).

## 4. Discussion

### 4.1. Is IRT a Good Tool to Evaluate the Effects of Milking on the Teats?

The teat temperature was significantly (*p* < 0.05) reduced at the global teat level before and after milking.

This result suggests that normal milking without teat liner slippage, aggressive machine settings, and abnormal over-milking, as observed on these three farms, could reduce the teat temperature in Alpine goats. The reason for this could firstly be the warm milk discharge, as the teat wall in Alpine goats is very thin, and the glandular cisterns have also lost temperature without any interaction with the milking equipment after milking [33]. Secondly, this reduction in temperature could be related to a possible slight reduction in blood flow in the teats due to the constriction at the base of the teat by the mouthpiece lip of the liner. 

The teat shape can vary in length and diameter in dairy cows and ewes, so the milking equipment must sometimes be adapted to the different breeds. In our goats, the teat shapes were quite variable, with four main classes representing the most important morphology, but many intermediate forms are possible. It seems very difficult to find a teat liner and teat that are perfectly adapted to each of these four shapes. This indicates a higher risk of problems at the liner and teat interface. This could be another reason for possible teat aggression during mechanical milking, which we have observed in our breeding and on our farms. We recommend homogenizing the animals in each herd to allow better adaptation of the cluster characteristics to the herds. Despite the enormous differences between the animals, the effect of teat shape never proved to be significant, but the interaction between teat shape and teat area is. The main difference was at the level of the teat cups, and the long cylindrical teats seemed to be warmed a little by the milking equipment. The liners could heat the tissue by friction because these long and thin cylindrical teats are the ones that move the most during milking, while the globular, conical teats completely fill the liner and only move at the end of milking when they empty, fold, and are sucked back into the liner by the vacuum rise when the milk flow has stopped [34]. The tip of the teat was always colder than the other parts of the teat, indicating an interaction with the external temperature and possibly due to a lack of blood circulation at this extremity, which is often exposed to vacuum fluctuations and more congestion [35]. The teat base area was also colder than the teat barrel area, which is consistent with the frequent observation of constriction rings at the teat base. 

Suggesting a better teat shape for milking machines remains difficult. The observation of constriction rings or redness or whiteness due to friction or constriction at the level of the mouthpiece seems to be negative for the conical shape and the widest globular teats and in favor of the more classic cylindrical shapes. Nevertheless, the longer cylindrical teats must be discarded because of the stronger interaction with the liner shown here and also because of the frequent marks of the short milk tube around the teat orifice. This phenomenon occurs when teats are too long and come under the buckling plan to touch the bottom of the teat. IRT does not help much to define the better teat shapes. 

In the only study conducted on goats by Alejandro et al., 2014, milking, on the contrary, led to a warming of the teat skin. The global settings of the machine were the same, but we have no information on over-milking, teat liner shape, and buckling pressure, which could greatly alter the results, as shown in cows [29,31]. Another hypothesis could be that the Murciano–Granadina goats, which are known to have lower milk production (in terms of volume) and therefore may have better-shaped udders with thicker teat skin, may be less affected by internal milk temperature emptying. There is no bibliographical data on this point, but the Alpine goat breed is known to have a very thin cistern wall so that the milk can sometimes seep through the skin. Sometimes “weeping teats” and “cystic dilatation of the teat cavities” are reported [36]. The wall at the teat base of our goats can be very thin, and milk can sometimes pass through it or accumulate subcutaneously between the skin folds, creating a milk cyst or teat/cistern hernia that can grow and later interfere with liner placement (Figure 2). In this way, our measurement of the effects of milking machines might be mainly contaminated by milk evacuation, while in Murciano–Granadina the sawn effect might better reflect the effects of the milking machines.

Nevertheless, dairy sheep with thicker teat and udder walls than goats, like ours, showed a decrease in teat temperature after milking using a similar evaluation method [32]. They described a greater drop in temperature with increased vacuum, which a change in blood circulation congestion in the teat could explain. It is, therefore, possible that the mean vacuum that is now recommended and used in France (around 40 KPa for the Low Line installation, 42 for the High Line) is too high according to our goats’ teat and udder structure. The teat end reaction (thickness change) measured during milking with the cutimeter could confirm this point. Regarding the data collected from the cows, it is important to emphasize that milking hygiene is generally performed by dipping or spraying products for asepsis before milking or by directly applying a wet cloth with tempered water and soap, followed by rapid drying. With these methods, the teat temperature on the teat surface drops rapidly due to the evaporation of the remaining water [29]. Depending on the time at which the temperature was measured after the removal of the milking cluster, the authors were able to detect a warming of the teat that was due to the return to its normal condition rather than to the influence of machine milking. This is probably the reason for the very large temperature variations recorded by these authors (1 to 2 °C), while we had only measured a cooling of less than 1.06 °C at the maximum at the teat orifice.

To summarize, IRT technology was capable of measuring the effects of milking machines in goats but needed to be adapted to the breed under study. We have detected global cooling, but it was likely that milk temperature masks the effects of the milking machine, which, when acting on some teat barrels, leads to a warming of the teat skin but is never sufficient to reverse the effect of milk temperature. Nevertheless, this technique was sensitive enough to measure the temperature difference in the teat skin due to the change in local blood circulation and could, in the future, help to better adapt the milking machine to the animals and improve animal welfare. Work must still be done to find the best combination between teat skin temperature and the more efficient and less aggressive milking process.

### 4.2. Can an Unbalanced Udder Alter IRT Responses to Milking?

Our study revealed a very high percentage of morphologically and functionally unbalanced udders (40 to 45%) in three of our farms. This confirms the initial observations made on this point in 15 other flocks in the main French production region (Vendée department) [37], with about 30% functionally unbalanced udders in flocks. The genetic origin of this problem could explain the fact that this percentage is overall independent of parity. It is very problematic when this high percentage is even observed in primiparous animals. Another reason for this lack of relation with parity could also be the culling of the more problematic goats between lactations. This illustrates an inadequate quality of udder attachment and internal structure in our French dairy goats, probably related to their rapid increase in milk yield over the years. This must be taken into account in future genetic selection. Since the 2018 campaign, French genetic selection companies have started to evaluate the associated morphological traits, which are also linked to the productive lifespan of goats in herds. Systematic recording of functional traits (milk flow curves showing steps during the declining phase) might also be of better help in the future [37] after systematic modeling and classification of milk flow curves as proposed by Legris et al. [38]. Our temperature measurements confirm the increased deleterious effect directed towards the smaller half of the udder and offer a potential explanation for the observed increase in inflammation in French goats in recent years (+675,000 cells/mL in Saanen and +485,000 cells/mL in Alpine breeds [39]). This imbalance contributes to an overmilking of the smaller half of the udder, increasing teat temperature. This increase partially compensates for the drop in temperature caused by milk let-down. Consequently, we observed a non-significant temperature variation on the teat of the smaller udder half, while there is a significant variation persists on the teat of the larger udder half. This aggression manifests itself mainly in the tubular part of the teat, as shown by the significant interaction between the effects of imbalance class and teat area. The significant (*p* < 0.05) interaction between balance and teat area suggests greater heating of the teat at the level of the barrel in small sides of unbalanced glands, which could be explained by probable over-milking of these half-udders within the goat.

### 4.3. Is the IRT Able to Differentiate the Degree of Mastitis? 

The youngest animals classically had a lower SCC score and produced significantly (*p* < 0.05) less milk, which generally leads to shorter milking times. This could explain why the teats of younger animals cool down better after milking and why the massaging effect of machine milking and the warming effect is less than in older goats with higher milk production. Interestingly, the shape of the teats also seems to be related to the SCC level, with SCC values being higher in globular teats and lower in long and cylindrical teats, while the other shapes are in between. Since we could not find a clear effect of milking on the different teat shapes, this influence of teat shape could be better explained by other risk factors such as the volume of teats, the risk of touching the legs, etc. 

The thermographic measurement showed a significant (*p* < 0.05) and positive regression between the IRT values and the SCC, as has already been shown by numerous authors, at least in sheep, cows, or camels [3,13,40]. This could indicate that the inflammation detected via the SCC is at least partially characterized by the clinical sign “calor” (increased heat), even if no “dolor” (pain), “tumor” (swelling) and “rubor” (redness) and “functio laesa” (loss of function), as the last four cardinal signs of inflammation [41], were observed. Nevertheless, due to the low value of the regression coefficient and the shape of the data distribution (point cloud), it seems difficult to make a real prediction for on-farm use despite the very wide range of SCC (from 5 × 10^3^ to 10 million cells/mL in our sample) found in goats compared to cows and sheep [42]. The regression coefficient was low, both with the pre-milking temperature and the temperature difference due to milking, indicating a lack of relation between machine action and udder inflammation.

Thus, IRT in goats, even with very high levels of inflammation generally associated with infection with minor and major pathogens [43], is not a good tool for predicting intra-mammary infection (IMI) compared to cows and ewes, probably due to many other physiological and environmental factors that may increase SCC in uninfected or already infected glands [17,26,44,45]. We confirmed this interpretation, as the timing of milking significantly (*p* < 0.05) changed the temperature before and after milking, with lower temperatures during morning milking, suggesting a greater influence of lower external temperature and/or internal metabolism after night rest in the herds. This effect could be greater in goats due to their particular udder shape, which is more pendulous and further away from the abdomen than in other animal species. The clinical signs of mastitis are generally mild to undetectable, and the IRT could not help us in the early detection of goats with mastitis, regardless of the significance of the infection, and could not be used for prophylactic purposes in goats. We confirmed observations in Murciano–Granadina goats [15], which found no differences in udder surface temperature before and after milking depending on health status, with aseptic glands having similar udder temperatures to detectably infected glands. 

## Figures and Tables

**Table 1:** Influence of milking on the temperature fluctuations of different teat areas independent of teat shapes in goats (LSmeans ± SEM; n = 4406).

Teat Areas	Temperature Difference ^1^ °C
Teat basis	−0.63 ± 0.05 ^b^
Teat barrel	−0.37 ± 0.05 ^c^
Teat end	−1.06 ± 0.05 ^a^
Total teat area	−0.61 ± 0.05 ^b^

^1^ Temperature difference = temperature after milking−temperature before milking. ^a,b,c^ Different letters in the same column indicate significant differences between the rows (*p* < 0.05).

**Table 2:** Influence of milking on the temperature variations of different teat areas and teat shapes in goats (LSmeans ± SEM; n = 4406).

	Temperature Difference ^1^ °C			
Teat Areas	Teat Shapes			
	Conical	Globular	Tubular Short	Tubular Long
Teat basis	−0.50 ± 0.09 ^b^	−0.52 ± 0.09 ^b^	−0.81 ± 0.09 ^b^	−0.67 ± 0.14 ^b^
Teat barrel	−0.47 ± 0.09 ^b^	−0.32 ± 0.09 ^b^	−0.58 ± 0.09 ^c^	−0.13 ± 0.14 ^c^
Teat end	−0.93 ± 0.09 ^a^	−1.07 ± 0.09 ^a^	−1.13 ± 0.09 ^a^	−1.10 ± 0.14 ^a^
Total teat area	−0.62 ± 0.09 ^b^	−0.53 ± 0.09 ^b^	−0.79 ± 0.09 ^bc^	−0.49 ± 0.14 ^bc^

^1^ Temperature variation = temperature after milking−temperature before milking. ^a,b,c^ Different letters in the same column indicate significant differences between the rows (*p* < 0.05).

**Table 3:** Influence of milking on the temperature variations of different teat areas in goats of different parity (LS means ± SEM; n = 4406).

	Temperature Difference ^1^ °C		
Teat Areas	Parity		
	1	2	≥3
Teat basis	−0.90 ± 0.10 ^Ab^	−0.60 ± 0.09 ^ABb^	−0.38 ± 0.08 ^Bb^
Teat barrel	−0.84 ± 0.10 ^Ab^	−0.37 ± 0.09 ^Bc^	0.09 ± 0.09 ^Cc^
Teat end	−1.42 ± 0.10 ^Aa^	−1.15 ± 0.09 ^Aa^	−0.61 ± 0.08 ^Ba^
Total teat area	−0.99 ± 0.10 ^Ab^	−0.62 ± 0.09 ^Ab^	−0.21 ± 0.08 ^Bb^

^1^ Temperature difference = temperature after milking−temperature before milking. ^a,b,c^ Different letters in the same column indicate significant differences between the rows (*p* < 0.05). ^A,B,C^ Different letters in the same row indicate significant differences between the columns (*p* < 0.05).

**Table 4:** Influence of milking on the temperature variations of different teat areas in goats with balanced and unbalanced udders (LS means ± SEM; n = 4406).

	Temperature Difference ^1^ °C	
Teat Areas	Unbalanced	Balanced
Teat basis	−0.62 ± 0.08 ^b^	−0.63 ± 0.06 ^b^
Teat barrel	−0.31 ± 0.08 ^c^	−0.44 ± 0.06 ^c^
Teat end	−1.10 ± 0.08 ^a^	−1.02 ± 0.06 ^a^
Total teat area	−0.57 ± 0.08 ^b^	−0.65 ± 0.06 ^b^

^1^ Temperature difference = temperature after milking−temperature before milking. ^a,b,c^ Different letters in the same column indicate significant differences between the rows (*p* < 0.05).

**Table 5:** Influence of farm, teat shape, and parity on logSCC (cells/mL) in goats (LS means ± SEM; n = 4406).

	Mean Log SCC			
Teat Shapes	Conical	Globular	Tubular Short	Tubular Long
	5.75 ± 0.01 ^b^	5.80 ± 0.01 ^a^	5.70 ± 0.01 ^bc^	5.65 ± 0.02 ^c^
**Farms**	**1**	**2**	**3**	
	5.57 ± 0.01 ^a^	5.61 ± 0.01 ^a^	5.99 ± 0.01 ^b^	
**Parity**	**1**	**2**	**≥3**	
	5.46 ± 0.01 ^a^	5.72 ± 0.01 ^b^	5.98 ± 0.01 ^c^	

^a, b, c^ Different letters in the same row indicate significant differences between the columns (*p* < 0.05).

## Data Availability

Data are contained within the article.
